# Nutrient Characteristics in Relation to Plant Size of a Perennial Grass Under Grazing Exclusion in Degraded Grassland

**DOI:** 10.3389/fpls.2018.00295

**Published:** 2018-03-12

**Authors:** Zhiying Liu, Taogetao Baoyin, Junjie Duan, Guofeng Yang, Juan Sun, Xiliang Li

**Affiliations:** ^1^Key Laboratory of Grassland Ecology, School of Ecology and Environment, Inner Mongolia University, Hohhot, China; ^2^National Forage Improvement Center, Institute of Grassland Research, Chinese Academy of Agricultural Sciences, Hohhot, China; ^3^School of Life Sciences, Qingdao Agricultural University, Qingdao, China; ^4^School of Animal Science and Technology, Qingdao Agricultural University, Qingdao, China; ^5^Key Laboratory of Grassland Ecology and Restoration of the Ministry of Agriculture, Hohhot, China

**Keywords:** phenotypic plasticity, livestock grazing, *Leymus chinensis*, plant nutrient strategy, functional traits

## Abstract

Identifying the linkages between nutrient properties and plant size is important for reducing uncertainty in understanding the mechanisms of plant phenotypic plasticity. Although the positive effects of grazing exclusion on plant morphological plasticity has been well documented, surprisingly little is known about the relationship of nutrient strategies with plant shoot size after long-term grazing exclusion. We experimentally investigated the impacts of grazing exclusion over time (0, 9, 15, and 35 years) on the relationships of nutrient traits (nutrient concentration, allocation, and stoichiometry) of with morphological plasticity in *Leymus chinensis*, which is a dominant species in grasslands of Inner Mongolia, China. Our results showed that there was a significantly negative correlation between the degrees of plasticity and stability of various morphological traits. Increases in plant size by 126.41, 164.17, and 247.47% were observed with the increase of grazing exclusion time of 9, 15, and 35 years, respectively. Plant size was negatively correlated with nitrogen (N) and phosphorus (P) concentrations, but was positively correlated with carbon (C) concentration. Biomass partitioning and leaf to stem ratios of nutrient concentrations contributed more than 95% of the changes in N, P, and C allocation in *L. chinensis* leaves and stems induced by grazing exclusions. Nine years’ grazing exclusion rapidly changed the nutrient concentrations (averaged by -34.84%), leaf to stem nutrient allocations (averaged by -86.75%), and ecological stoichiometry (averaged by +46.54%) compared to free-grazing, whereas there was no significant trend of these nutrient traits across the 9, 15, and 35 years’ grazing exclusion in *L. chinensis* individuals. Our findings suggest that with the increase of the duration of the grazing exclusion, time effects on plant performances gradually weakened both in plant morphological plasticity and nutrient properties. There is a significant negative effect between plant sizes and nutrient traits under long-term grazing exclusion.

## Introduction

Phenotypic plasticity is defined as the ability of a single plant genotype to produce different phenotypes (morphology, phenology, or physiology) under various biotic and abiotic disturbances, including herbivore grazing, bio-invasion, elevated CO_2_, warming, and drought (Nicotra et al., 2010; Dostál et al., 2016; Wang D. et al., 2017). The potential role of phenotypic plasticity on the change of ecosystem function has attracted the interest of grassland ecologists. Furthermore, it provides a new theoretical perspective for understanding how plant functional traits mediate the restoration of grassland ecosystem performance under grazing exclusion (Li et al., 2016a; Wang D. et al., 2017).

In grazing ecosystem, the grazing of large herbivores directly influences the phenotypic characteristics of grassland species by impacting leaf photosynthesis, respiration, and plant development; it can also influence indirectly grassland species by changing the soil micro-environment (Ren et al., 2017; Abdalla et al., 2018). It has long been recognized that plant individuals will become dwarfed in response to overgrazing, whereas plant size will return to normal after exclusion from grazing over a multi-year period (Armitage et al., 2012). Recent results from a long-term grassland trial indicated sheep grazing caused the decline of aboveground biomass of *Leymus chinensis*, primarily through a ‘bottom-up’ effect due to the asymmetrical response of different plant functional traits with the variations in individual plant size (Li et al., 2015a). During grazing exclusion, however, the patterns of the phenotypic variations of plant species in a long-term study in a degraded pasture are largely unknown.

Over the past few decades, grazing exclusions have become a primary management practice for the restoration of degraded pastures in semi-arid regions worldwide (Han et al., 2008; Jing et al., 2014). Plant–soil interactions play an important role in the maintenance of both plant community composition and soil properties in the transition from degraded grasslands to restored grasslands by grazing exclusion (Laliberté et al., 2012; Odriozola et al., 2017). In general, grazing exclusion can enhance ecosystem stability and productivity in the initial years of the exclusion. (Deng et al., 2017). However, some studies have demonstrated that long-term grazing exclusion could exert a negative influence on species renewal and productivity (Silcock and Fensham, 2013). For example, Jing et al. (2014) reported that the productivity and biodiversity were higher in the mid- and transitional stages of succession, but decreased with increasing restoration time in a semiarid grassland.

Plasticity in plant nutrient traits, including the content, resorption, stoichiometry, and allocation of nutrients, is predicted to play a key role in regulating the morphological plasticity, indirectly affecting the carbon (C) cycle and ecosystem stability (Lü et al., 2012a; Heyburn et al., 2017). The plasticity in the nutrient resorption efficiency and proficiency is an important nutrient strategy for plants subjected to grazing (da Silveira Pontes et al., 2010). In a semi-arid grassland, Lü et al. (2015) found that the dominate grass species displayed decreasing nutrient resorption with the duration of grazing exclusion for nitrogen (N) and phosphorus (P). There is abundant evidence that C:N:P stoichiometry in plant tissues are generally associated with plant growth strategies, which strongly influence the capacity for adaptation to grazing disturbance (Velthuis et al., 2017; Yang et al., 2017). Moreover, nutrient allocation is regulated by grazing primarily through the changes in biomass partitioning and nutrient concentrations (Li et al., 2016b). Grazing-induced shifts in nutrient allocation patterns among different tissues are indirectly influenced by the availability of soil resources (Bai et al., 2012; Zheng et al., 2012). However, there is a lack of long-term investigations into the effects of grazing exclusion on the relationship between plant morphological plasticity and the nutrient strategies of grassland plants.

Native pastures in typical steppe regions cover the largest area in the eastern Eurasian temperate grasslands at the northern boundary of China (Schönbach et al., 2009). In these pastures, the dominant grass species is *L. chinensis*, which grows rapidly and has a high tolerance to arid conditions. This perennial rhizomatous grass is also very palatable to grazing animals and has a high forage value (Ma et al., 2014; Ren et al., 2014). *L. chinensis* is considered one of the most promising grass species for grassland rehabilitation and restoration in arid regions of northern China (Liu and Han, 2008; Huang et al., 2015).

Therefore, in this study, *L. chinensis* (the dominant grassland species) was chosen as a model plant for exploring ecological processes. We examined the effects of grazing exclusion on morphological plasticity, nutrient concentration, accumulation, allocation, and ecological stoichiometry of *L. chinensis* along a time series in a degraded pasture located in Xilinhot, Inner Mongolia, China. Specifically, we addressed the following four questions: (i) How do morphological plasticity and biomass allocation change across a grazing exclusion chronosequence? (ii) How does grazing exclusion influence the relationships between the size of *L. chinensis* and the concentrations of carbon (C), nitrogen (N), and phosphorous (P) in leaves and stems? (iii) How does grazing exclusion affect C, N, and P allocation strategies in *L. chinensis* in plants of varying sizes? (iv) How does grazing exclusion affect the relationship between *L. chinensis* C:N:P stoichiometry and morphological plasticity?

## Materials and Methods

### Study Area

This study was conducted at the Xilingol Grassland Ecosystem Observation and Research Station (43°38′ N, 116°42′ E) in the Xilin River catchment area of Inner Mongolia, China. The site has an altitude of ∼1,200 m a.s.l. and a semiarid continental climate characterized by a mean annual (1979–2013) precipitation of 328.54 mm and a mean annual temperature of 0.89°C. Typically, maximum precipitation coincides with the months with the highest temperatures in June, July, and August. The growing season for perennial plants lasts for approximately 150 days from May to September. The vegetation in this region mainly consists of herbaceous plants, such as *L. chinensis, Stipa grandis, Achnatherum sibiricum, Agropyron cristatum, Caragana microphylla*, and *Cleistogenes squarrosa*. The perennial rhizome grass *L. chinensis* dominates the typical steppe communities in Eurasian steppe regions, especially in the Xilingol Grassland (He et al., 2009; Li et al., 2016a). The major soil types found in this grassland are calcic chestnuts and calcic chernozems. The content of calcium carbonate accounts for 0.80% of the total soil in topsoil layers (He et al., 2009).

### Site Description and Experimental Design

Three vegetation restoration treatments and one free-grazing treatment in *L. chinensis* grassland were conducted in this study. The long-term restoration treatments were excluded from grazing or were enclosed by fencing, with each plot closed off in different years. The chronosequence of grazing exclusion was represented by three nearby plots, which included grazing exclosures dating from 2005, 1999, and 1979. Therefore, the duration of grazing exclusion of the four sites was 0, 9, 15, and 35 years to the sampling year of 2014. Each plot ranged in size from 5 to 40 ha. Like most chronosequence studies, the sites were subjected to pseudo-replication and ‘space-for-time substitution’ approaches to overcome the obstacle of studying up to several decades of grazing exclusion (Conant and Paustian, 2002; Lü et al., 2015; Eby et al., 2017). The four experimental plots, the three restored sites and one grazed contr ol site, were contiguously distributed on the same upper basalt platform; they appeared to be topographically and floristically similar and generally experienced low frequency and severity of disturbance, thus grazing exclusion was identified as the only important factor of change across the plots.

The grazing plot (control plot) had been grazed by sheep and goats year-round for more than 50 years at a stocking rate of approximately 3 sheep per hectare. This was significantly higher than the local stocking rates of 1.5 sheep per hectare needed to maintain grass-livestock balance, as recommended by the local government. Livestock consumed an estimated more than 75% of the aboveground biomass each year and the vegetation cover during our fieldwork was 25∼30%. The living and litter biomass of the degraded community were only in approximately 50 and 5 g⋅m^-2^, respectively. Though the palatable grass of *L. chinensis* generally dominated in native community, it only accounted for approximately 10% of the total aboveground productivity in the degraded grassland induced by livestock selective grazing (Li et al., 2015a). The grazing plot had a nutrient-poor topsoil characterized by total N of 1.8 g kg^-1^, total P of 0.3 g kg^-1^, inorganic N of 3.6 mg kg^-1^ and available P of 5.0 mg kg^-1^ (Lü et al., 2015). The topsoil had an organic matter content of 2.0%.

Grazing exclusion plots (enclosed 9, 15, and 35 years) were located adjacent to the grazing plots, ranging in aboveground biomass from 140 to 160 g m^-2^. According to our community monitoring data, the relative contribution of *L. chinensis* to total above-ground biomass obviously increased from about 10% (grazing plots) to 60 % (1979 enclosure) with the duration of grazing exclusion in the chronosequence. The bulk density (1.2 g cm^-3^) of topsoil was always significantly lower than the free-grazing plot. However, the topsoil nutrients in 9, 15, and 35 years’ plots were found in relatively high levels (total N of 2.2∼2.6 g kg^-1^ and total P of 0.3 g kg^-1^, inorganic N of 5.0∼9.2 mg kg^-1^ and available P of 3.1∼5.4 mg kg^-1^) (Lü et al., 2015). The humus layer thickness of was in the range of 30∼45 cm, and had an organic matter content of 2.2∼2.5%.

In early spring in 2014, we established three 15 × 15 m reduplicated sub-plots representative of the vegetation present within each plot of distinct grazing exclusion history. Five 1 m × 1 m quadrats were established in each sub-plot for sampling, with a total of 15 quadrats in each treatment. In each grazing quadrat, a temporary movable exclosure cage was set up at each sampling point prior to grazing and before the growing season began in the early spring of 2014. The quadrats were at least 5 m apart and the physiographic conditions within the quadrats were similar.

### Sampling and Measurement

Field sampling was conducted from August 15th to 18th, 2014, which corresponds with the annual peak standing biomass. Five *L. chinensis* individuals were selected randomly in each 1 m × 1 m quadrat. Phenotypic functional traits (plant height, leaf number, leaf length, leaf width, stem length, stem diameter, and biomass accumulation) of each *L. chinensis* individual were measured in the laboratory after the entire aboveground portion was completely clipped in the field. Leaf width and stem diameter were measured using vernier calipers (precision = 0.01 mm). After the measurement of phenotypic traits, the leaves and stems were packaged in individual paper bags, and all plant samples were oven-dried at 65°C for at least 48 h. Stem, leaf, and total biomass of *L. chinensis* were measured after drying.

The leaves and stems of the *L. chinensis* individuals in each quadrat were combined, pulverized using a mechanical mill (Retsch MM 400, Retsch GmbH & Co KG, Haan, Germany), and passed through a 40-mesh sieve. The total concentration of C in leaves and stems was determined using the H_2_SO_4_-K_2_Cr_2_O_7_ oxidation method. Subsamples were digested in H_2_SO_4_-H_2_O_2_ (Bennett et al., 2003). The total concentration of N was analyzed using an Alpkem autoanalyzer (Kjektec System 1026 Distilling Unit, Sweden), and the total concentration of P was measured colorimetrically at 880 nm after reaction with molybdenum blue. All stoichiometric ratios of C:N:P were reported as mass ratios (Lü et al., 2012b).

### Statistical Analysis

The mean of each of the plant traits was calculated from measurements taken from all the *L. chinensis* individuals in a single quadrant. A principal component analysis (PCA) was performed to determine relationships among plant traits and the effect of grazing exclusion on these traits (Fort et al., 2015; Li et al., 2015b). For this analysis, we centered and normalized all variables with their standard deviations because they had different units. The importance of a trait in a given component is indicated by its relative loading on a component. Responses of functional traits of *L. chinensis* in the grazed and enclosed grassland plots were analyzed using the plasticity index (*PI*) (Olmo et al., 2014; Li et al., 2015a):

(1) $$\begin{array}{c} PI=\frac{FE-FG}{FE} \end{array}$$

where *FE* is the value of functional traits in habitats of grazing exclusion plots, and *FG* is the value of functional traits in long-term grazed habitats.

The degrees of stability of *L. chinensis* traits in the sampled populations were analyzed by a stability index (*SI*), which was represented by the reciprocal of the coefficient of variation (*CV*):

(2) $$\begin{array}{c} SI=\frac{SD}{FM} \end{array}$$

where *SD* and *FM* are the standard deviation and the average values of all the functional traits in the grazing exclusion plots, respectively.

The Kolmogorov–Smirnov test was used to test for normality among the average plant phenotypic traits and C, N, and P concentrations in *L. chinensis* leaves and stems. For each *L. chinensis* individual, C, N, and P content were calculated by multiplying the C, N, and P concentrations by the biomass of the corresponding tissues.

Structural equation modeling (*SEM*) was used to analyze hypotheses that may explain the pathways responsible for the indirect effects of grazing exclusions on biomass accumulation and allocation, and C, N, and P accumulation of individual *L. chinensis* plants (Byrne, 2013; Li et al., 2016b). Structural equation models were developed based on hypothesized relationships between variables and tests of preliminary models. Co-varying factors with non-significant regression weights within the *SEM*s were not included. The final *SEM*s were applied to each of the *L. chinensis* functional trait indices. Occasionally, it was necessary to alter the relationship between a measured *L. chinensis* functional trait and the rest of the model (Li et al., 2016b). The utility of each functional trait index within the *SEM* was compared based on a number of measures. These include the power of the particular model to explain the variation (*r*^2^), measures of model significance and fit (*χ*^2^ and AIC), and the significance of the functional trait variables within the model (significance of regression weights) (Li et al., 2016a).

Significant differences in plant traits between various treatments were evaluated using one-way analysis of variance (ANOVA). Pearson’s correlation method was selected to test the relationships between two variables. All statistical analyses were performed using SPSS 19.0 statistical software (SPSS, Inc., Chicago, IL, United States), with *P* < 0.05 set as the level of significance. Structural equation modeling analysis was performed using AMOS 20.0 software (IBM, SPSS, Armonk, NY, United States).

## Results

### Morphological Plasticity

**FIGURE 1:**
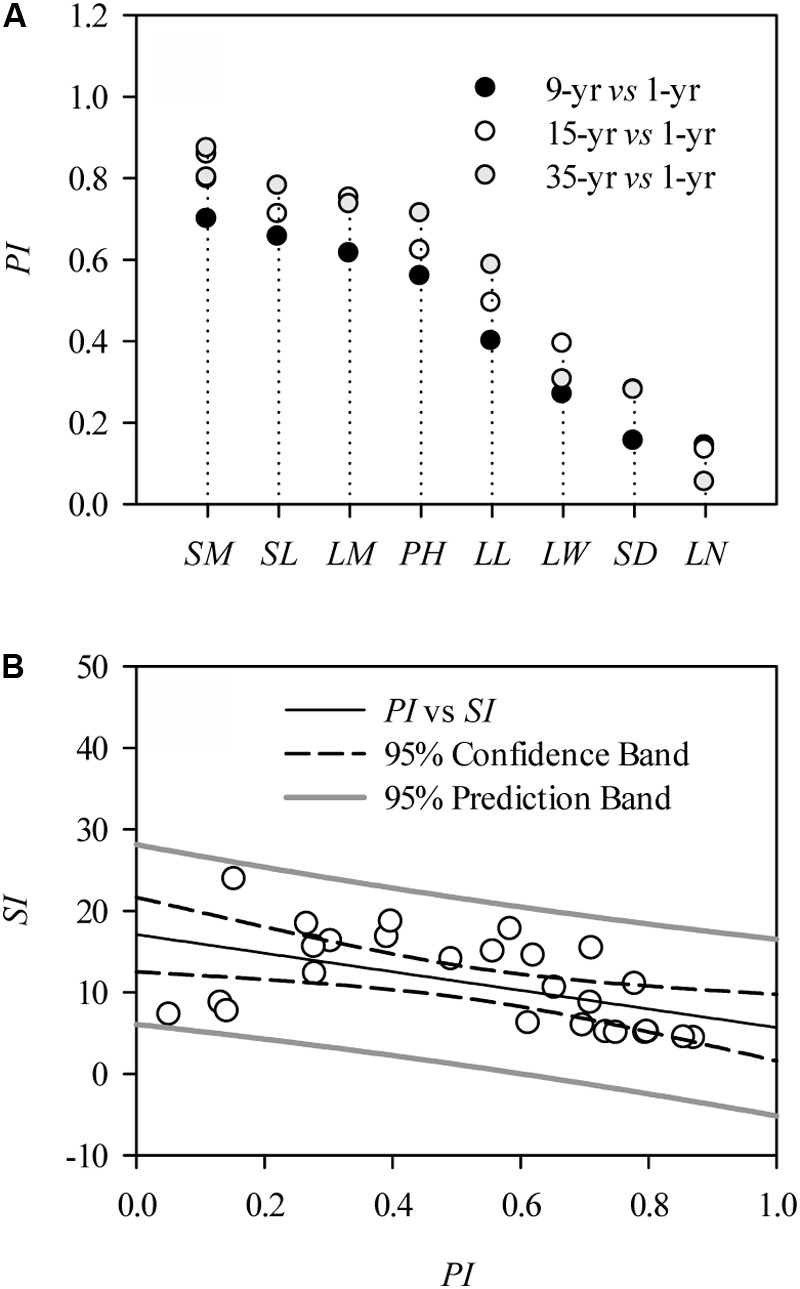
Morphological plasticity of *Leymus chinensis* in response to grazing exclusions. **(A)** Plasticity indexes (*PI*) of morphological traits, including: *SM*, stem mass; *AM*, aboveground mass; *SL*, stem length; *LM*, leaf mass; *PH*, plant height; *LL*, leaf length; *LW*, leaf width; *SD*, stem diameter; and *LN*, leaf number. **(B)** Correlation between *PI* and stability indexes (*SI*) of plant morphological traits of *L. chinensis* in response to grazing exclusion; the relation between them is fitted by a Pearson correlation (*r* = –0.52, *P* < 0.01).

At the individual level, the morphological traits, including leaf and stem traits, were significantly positively affected by 9, 15, and 35 years’ grazing exclusion (*P* < 0.05; **Table 1**). However, some *L. chinensis* traits, such as leaf number, leaf width, and stem diameter, in long-term grazing exclusions (the plot from 1979) were slightly lower compared to those in the plot established in 1999 (**Table 1**). Ranking of the highly varied plasticity indexes of morphological traits indicated that the morphological traits of *L. chinensis* can be distinguished into sensitive traits (i.e., leaf length) and sluggish traits (i.e., stem diameter) in response to grazing exclusion over time (**Figure 1A**). There was a significantly negative correlation between plasticity and stability indexes across grazing exclusions over time (*P* < 0.01; **Figure 1B**).

**Table 1:** Mean (and SD) of individual plant size of *Leymus chinensis* in response to grazing exclusion of differing lengths in a degraded pasture.

Categories	Indexes	CK		9-yr		15-yr		35-yr	
		Mean	*SD*	Mean	*SD*	Mean	*SD*	Mean	*SD*
	Plant height (cm)	23.89^d^	3.30	54.09^c^	3.62	63.11^b^	4.37	83.01^a^	5.41
	Leaf number	4.80^c^	0.56	5.60^a^	0.74	5.53^ab^	0.64	5.07b^c^	0.70
Plant	Leaf length (cm)	13.37^d^	1.14	22.23^c^	1.20	26.35^b^	1.88	32.24^a^	1.82
properties	Leaf width (mm)	5.41^d^	0.65	7.39^c^	0.40	8.90^a^	0.53	7.78^b^	0.48
	Stem length (mm)	1.46^c^	0.17	1.72^b^	0.07	2.02^a^	0.17	2.02^a^	0.13
	Stem diameter (mm)	10.92^d^	2.13	31.62^c^	3.02	37.69^b^	4.36	49.59^a^	4.53

*CK is the control; 9-yr is 9-year grazing exclusion; 15-yr is 15-year grazing exclusion; 35-yr is 35-year grazing exclusion. Different superscripts denote significant differences (*P* < 0.05).*

**FIGURE 2:**
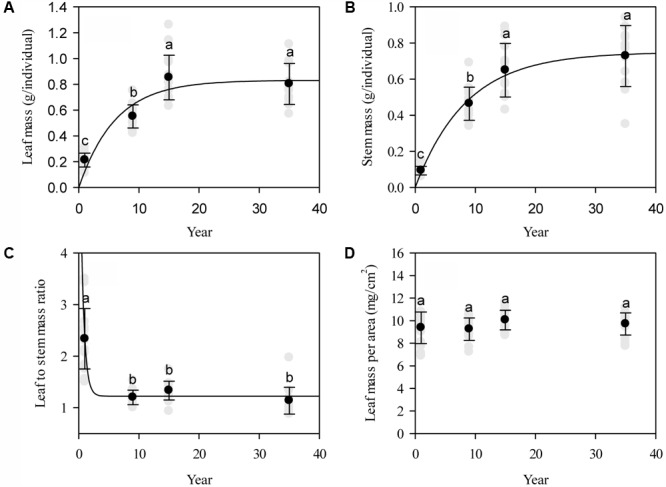
Patterns of individual biomass accumulation **(A)** and allocation (mean ± SD) in leaves and stems **(B–D)** of *Leymus chinensis* following grazing exclusion in a degraded pasture. The patterns of leaf mass (*r* = 0.85, *P* < 0.001) and stem mass (*r* = 0.90, *P* < 0.001) are fitted by an exponential rise to the maximum, whereas the leaf to stem mass ratio (*r* = -0.82, *P* < 0.001) displays an exponential decay. Different superscripts denote significant differences (*P* < 0.05).

**FIGURE 3:**
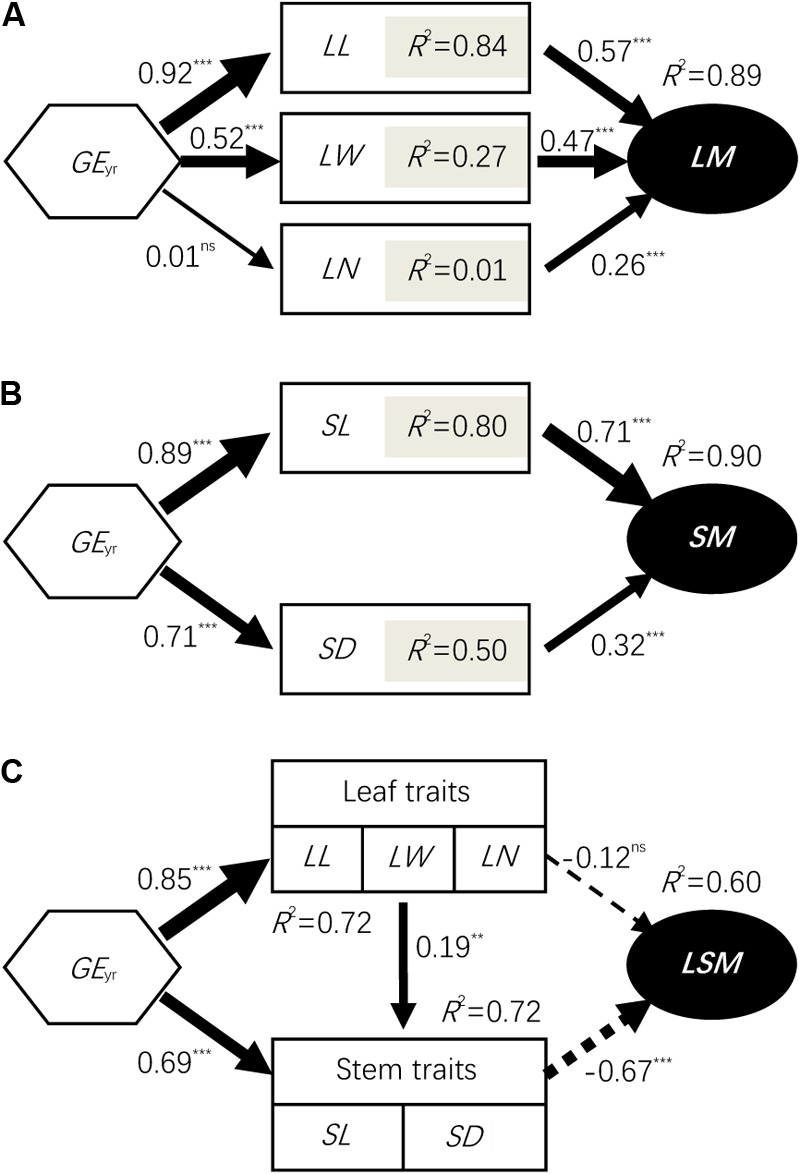
Structural equation modeling (*SEM*) results for the grazing exclusion effects on biomass accumulation **(A,B)** and allocation **(C)** at the individual level. Values associated with arrows represent standardized path coefficients. Solid and dashed arrows represent positive and negative paths, respectively, in a fitted SEM depicting the impact of variables on stem length decline. *R*^2^ values associated with response variables indicate the proportion of variation explained by relationships with other variables. Symbols: ^∗∗∗^represents *P* < 0.001; ^∗∗^represents *P* < 0.01; ns represents *P* > 0.05. *LM*, leaf mass; *SM*, stem mass; *LSM*, leaf to stem mass ratio; *AM*, aboveground mass; *PH*, plant height; *LL*, leaf length; *LW*, leaf width; *SL*, stem length; *SD*, stem diameter; and *LN*, leaf number; *GE*_yr_, number of years of grazing exclusion.

Affected by grazing exclusion, leaf mass per individual significantly increased (*P* < 0.01; **Figure 2A**). The variation of leaf mass accumulation per *L. chinensis* individual was primarily driven by the variation of leaf length, leaf width, and leaf number (*P* < 0.01; **Figure 3A**). Similarly, stem mass accumulation was highly correlated with the increase of grazing exclusion time, with an initial exponential increase (*P* < 0.01; **Figure 2B**). The variation of stem mass per *L. chinensis* individual was caused by the variation of stem length and stem diameter (*P* < 0.01; **Figure 3B**). The leaf-to-stem biomass ratio was negatively correlated with the increase of grazing exclusion time (*P* < 0.01; **Figures 2C,D**). We detected that the change in stem traits was the major driving factor for the decreased leaf-to-stem allocation induced by grazing exclusion (*P* < 0.01; **Figure 3C**).

### Tradeoff Between Nutrient Concentration and Accumulation

**FIGURE 4:**
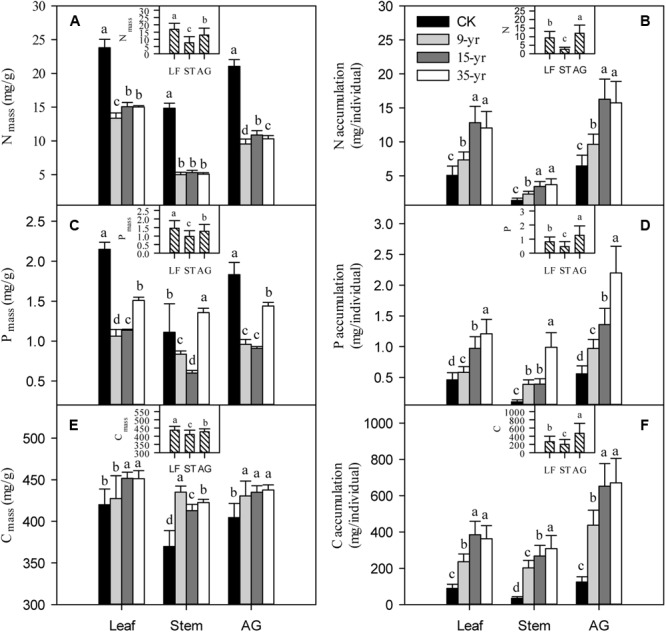
Effects of grazing exclusion on C, N, and P concentrations (**A,C,E**, respectively) and accumulations (**B,D,F**, respectively) in leaves, stems, and the aboveground portions of *Leymus chinensis* individuals following grazing exclusion (mean ± SD). CK is open grazing; 9-yr is 9-year grazing exclusion; 15-yr is 15-year grazing exclusion; 35-yr is 35-year grazing exclusion; *AG* is aboveground portion; *LF* is leaf; *ST* is stem. Different superscripts denote significant differences (*P* < 0.05).

Plant size was negatively correlated with N and P concentrations, but it was positively correlated with C concentration. However, with the increase of plant size, N, P, and C accumulations increased linearly (**Table 2**; *P* < 0.05). We found that there was a rapid decrease of N and P concentration in leaves, stems, and the aboveground portion of *L. chinensis* individuals after grazing was excluded from the degraded pasture (*P* < 0.05). However, only slight changes in N and P concentration were found across the exclusion pastures established in 2005, 1999, and 1979 (**Figures 4A,C**). The accumulation of N and P in leaves, stems, and the aboveground portion gradually increased with the increase of grazing exclusion time (*P* < 0.05; **Figures 4B,D**). In contrast, C concentrations rapidly increased shortly after grazing exclusion (*P* < 0.05), whereas it was not significantly affected by the increase of the grazing exclusion time (*P* > 0.05; **Figure 4E**). The trend of C accumulation following grazing exclusion was similar to the trends observed for N and P (*P* < 0.05; **Figure 4F**). In addition, we detected that the tradeoffs between the concentration and accumulation of N and P were similar (**Table 3**; *P* < 0.05).

**Table 2:** Relationships of plant size (PC axis 1) with C, N, and P concentrations (X_mass_) and accumulations (X_accumulation_) in leaves, stems, and aboveground portions in *Leymus chinensis* individuals.

Indexes	Leaf		Stem		Aboveground	
	*r*	*P*	*r*	*P*	*r*	*P*
N_mass_	–0.66	<0.01	–0.73	<0.01	–0.72	<0.01
P_mass_	–0.42	<0.01	0.32	<0.05	–0.27	<0.05
C_mass_	0.45	<0.01	0.63	<0.01	0.56	<0.01
N_accumulation_	0.59	<0.01	0.71	<0.01	0.63	<0.01
P_accumulation_	0.73	<0.01	0.90	<0.01	0.85	<0.01
C_accumulation_	0.66	<0.01	0.78	<0.01	0.73	<0.01

**Table 3:** Relations between C, N, and P concentrations and accumulations in leaves, stems, and aboveground portions in *Leymus chinensis* individuals.

Elements	Leaf		Stem		Aboveground	
	*r*	*P*	*r*	*P*	*r*	*P*
N	–0.54	<0.01	–0.68	<0.01	–0.61	<0.01
P	–0.31	<0.05	0.44	<0.01	–0.17	>0.05
C	0.58	<0.01	0.67	<0.01	0.66	<0.01

### Nutrient Allocation and Stoichiometry

**FIGURE 5:**
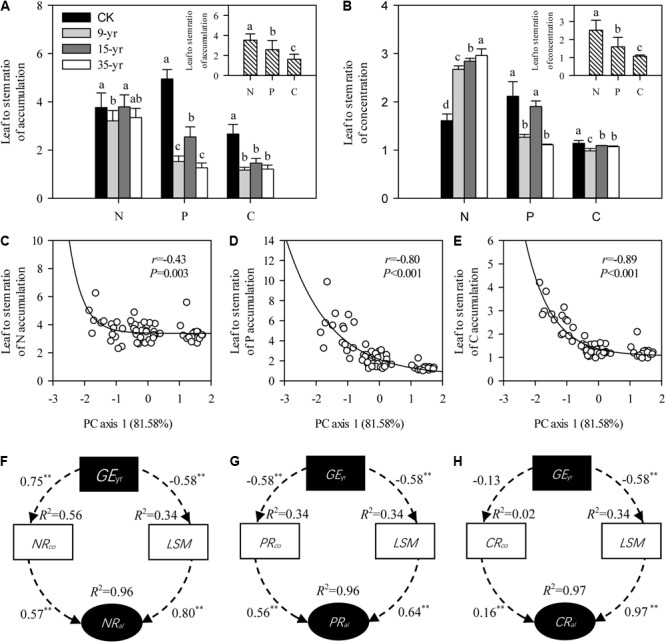
Effects of grazing exclusion on the N, P, C allocations in leaves and stems of *Leymus chinensis* individuals (mean ± SD). **(A)** Leaf to stem ratios of N, P, and C accumulation in response to grazing exclusions. **(B)** Leaf to stem ratios of N, P, and C concentration in response to grazing exclusions. **(C–E)** Relationships of plant traits (PC axis 1) with leaf to stem ratios of N, P, and C accumulation, respectively. **(F–H)** Pathways of the changes in leaf to stem ratios of N, P, and C accumulation induced by grazing exclusions, respectively. Values associated with arrows represent standardized path coefficients. *R*^2^ values associated with response variables indicate the proportion of variation explained by relationships with other variables. Symbols: ^∗∗∗^represents *P* < 0.001. *XR*_co_ is leaf to stem ratios of N, P, and C concentrations; *XR*_al_ is leaf to stem ratios of N, P, and C accumulations; CK is open grazing; 9-yr is 9-year grazing exclusion; 15-yr is 15-year grazing exclusion; 35-yr is 35-year grazing exclusion. Different superscripts denote significant differences (*P* < 0.05).

There was a rapid decrease in leaf to stem ratio of P and C accumulation in *L. chinensis* individuals after grazing exclusion (*P* < 0.05; **Figure 5A**), but no distinct trend was found in these ratios across the chronosequence (**Figure 5A**). In general, leaf to stem ratios of N, P, and C accumulation were negatively correlated with plant size variation induced by grazing exclusion, as indicated by the model of exponential decay (*P* < 0.01; **Figures 5C–E**). To explore the reason for changes in nutrient allocation, we further determined that there was an increase in the leaf to stem ratio of N concentration, but a decrease in P and C with a longer duration of grazing exclusion (*P* < 0.05; **Figure 5B**). The pathway analysis fitted by SEM showed that the changes in N, P, and C allocation induced by grazing exclusions were in part due to leaf to stem ratios of biomass and nutrient concentration (*P* < 0.01; **Figures 5F–H**).

**FIGURE 6:**
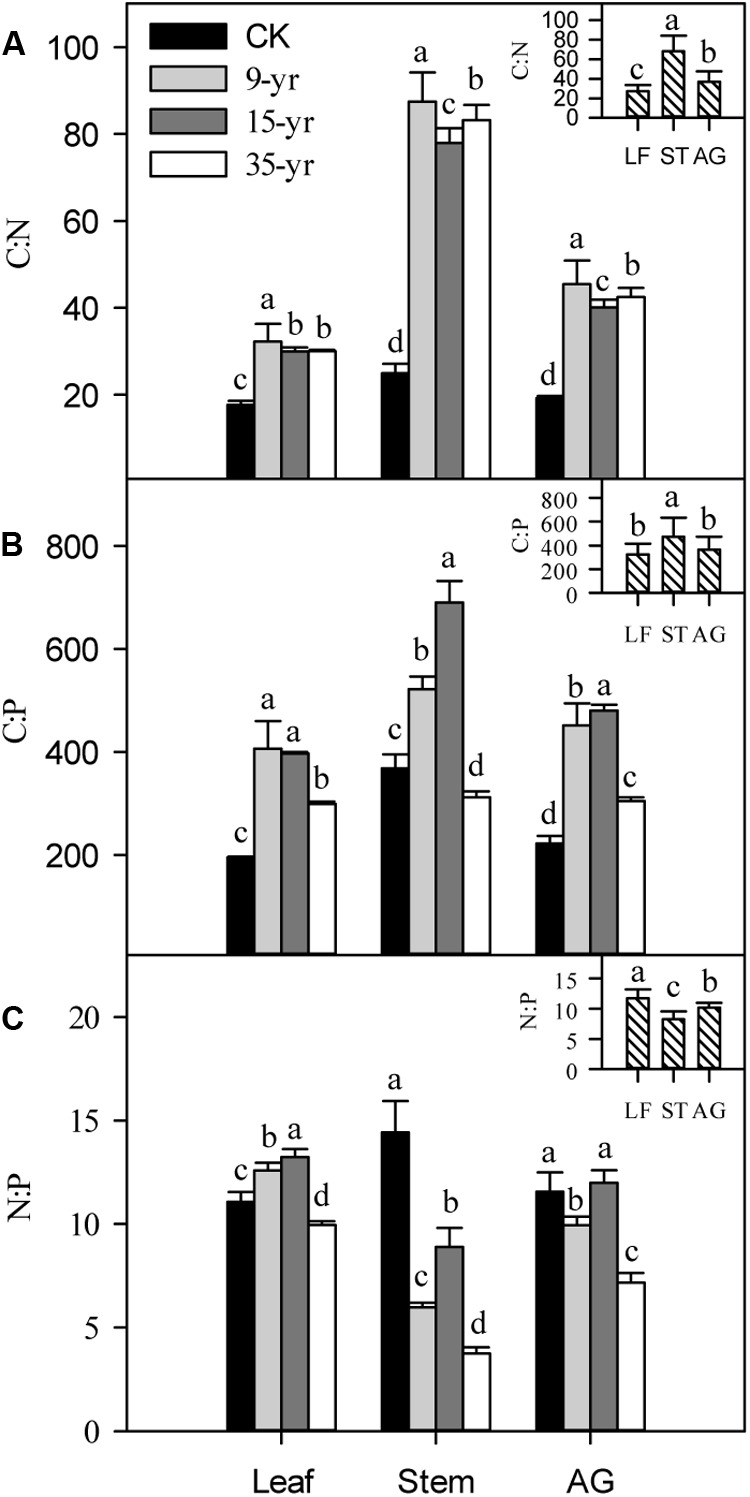
Effects of grazing exclusion on C:N **(A)**, C:P **(B)**, and N:P **(C)** in leaves (LF), stems (ST), and the aboveground (AG) portions in *Leymus chinensis* individuals following grazing exclusion (mean ± SD). Different superscripts denote significant differences (*P* < 0.05).

Grazing exclusion rapidly increased the C:N and C:P in leaves, stems, and the aboveground portions of *L. chinensis* individuals (*P* < 0.05; **Figures 6A,B**), but there was no significant trend across the three grazing exclusion chronosequence treatments (**Figure 6**). In addition, the N:P ratio gradually decreased in *L. chinensis* stems and aboveground portions growing in grazing exclusion plots (*P* < 0.05; **Figure 6C**). In contrast, leaf N:P increased initially and then decreased with the increase of grazing exclusion time (**Figure 6C**). Finally, C:N and C:P were positively correlated with plant traits, but N:P was negatively correlated with plant traits (*P* < 0.05; **Table 4**).

**Table 4:** Relationships of plant size (PC axis 1) with C, N, and P ecological stoichiometry in leaves, stems, and aboveground portions in *Leymus chinensis* individuals.

Elements	Leaf		Stem		Aboveground	
	*r*	*P*	*r*	*P*	*r*	*P*
C:N	0.63	<0.01	0.72	<0.01	0.69	<0.01
C:P	0.31	<0.05	–0.21	>0.05	0.17	>0.05
N:P	–0.38	<0.01	–0.81	<0.01	–0.80	<0.01

## Discussion

Morphological plasticity, which exists in various terrestrial plant species, is a comprehensive strategy to adapt to heterogeneous habitats. This strategy allows plants to effectively gain access to more resources (de Vries et al., 2016). Grazing affects natural pastures in the macro-scale performance of ecosystems and the plant function at the micro-scale in semi-arid regions (Sanjuán et al., 2017). Our results demonstrated that *L. chinensis* leaves and stems exponentially tended to be larger after grazing exclusion over time compared with plants in free-grazing conditions. This is consistent with the findings described in several previous studies (Deléglise et al., 2011; Wu et al., 2013). We detected a dramatic change (0.05–6.8) in the plasticity of different functional traits with the increase of grazing exclusion time. The sensitivity of stems was higher than that of leaves with the increase of grazing exclusion time, indicating that *L. chinensis* can exhibit rapid pant growth by promoting stem elongation. A previous study on leaf traits indicated that the strategies for stability and plant protection under grazing disturbance provides some indirect evidence for our findings (Jaurena et al., 2013).

In our study, individual *L. chinensis* leaf and stem mass accumulation exponentially increased during the peak-growth season with the increase of grazing exclusion time. This is consistent with results from previous studies conducted in a variety of grassland habitats (Courtois et al., 2004; Shrestha and Stahl, 2008). However, there was a slight decrease in biomass accumulation after long-term grazing exclusion (plot from 1979) compared to the medium-term plot (established in 1999). This may be related to the tradeoff between leaf and stem growth after long-term grazing exclusion (35-years in this study). Specifically, intraspecific and interspecific competition for light resources will be intensified in grazing exclusion habitats (Borer et al., 2014); therefore, plant stem elongation will be stimulated as a shade avoidance response to light limitations (Baldissera et al., 2016). Photoassimilates will be preferentially allocated to the stem to rapidly increase the plant individual height during the stem elongation process (Williamson et al., 2012), which may be the underlying mechanism for our finding that the leaf to stem mass ratio significantly decreases in the long-term grazing exclusion habitats.

We initially hypothesized that increased nutrient concentrations in plant tissues would support plant morphological plasticity in response to grazing exclusion, but this was not supported by our results that grazing exclusion significantly decreased N and P concentrations. Theoretically, this may be explained by three previously discussed hypotheses. First, the growth dilution hypothesis: if increased accumulation of shoot mass is more than the increase in nutrient acquisition under grazing exclusion, nutrient concentrations will decrease (Li et al., 2016b). Second, the decreasing metabolism hypothesis: decreased N and P concentrations might weaken the number and activity of photosynthetic enzymes and improve the photosynthetic rate when plants are grown under grazing exclusion conditions (Scogings et al., 2013). Third, the nutrient competition hypothesis: competition for soil nutrients under grazing exclusions became significantly more intense compared to grazed habitats, contributing to the decrease of nutrient concentrations (Lü et al., 2015). In general, our finding that grazing exclusion significantly decreased N and P concentrations in *L. chinensis* is similar with previous studies on experimental grazing, mowing, elevated CO_2_, and drought, supported by the mechanisms discussed above (Fernando et al., 2014; Li et al., 2016b).

We observed a sharp decrease of N and P concentrations after grazing exclusion in the degraded pasture; however, only minor changes in N concentration and increases in P were detected across grazing exclusions established in 2005, 1999, and 1979. Therefore, the growth dilution hypothesis provides only a partial explanation for these findings. It is also likely associated with the drivers of the metabolism regulation hypothesis. In contrast, we found that C concentrations in the three sampling positions rapidly increased after grazing exclusions. This is most likely related to greater photosynthetic function and an adequate supply of carbon assimilates in the grazing excluded pastures. This suggests that there was a tradeoff of C concentration with N and P concentrations, and it may provide new insight into plant nutrient strategies in response to grazing exclusion.

An interesting result was that leaf to stem ratios of N, P, and C accumulation were negatively correlated with plant size induced by grazing exclusion. We also found that the degrees of plasticity of *L. chinensis* individuals in stem traits were markedly higher than that in leaf traits under grazing exclusion. We speculate that there may be some theoretical correlation between nutrient allocation and leaf-stem allometry (Li et al., 2015a). Our SEM results demonstrated that the changes in N, P, and C allocation induced by grazing exclusion were derived from leaf to stem ratios of biomass and nutrient concentration. A previous study has shown that light competition affects plant growth in restored grassland communities (Loydi et al., 2015). For example, shade avoidance can significantly promote the nutrient allocation and growth of plant stems (Heger, 2016). Hence, with the increase of grazing exclusion time, the increased stem nutrient allocation in response to light competition may explain the observed stimulation of stem elongation and biomass accumulation.

The C:P and C:N ratios of plant illustrate the C assimilation efficiency when absorbing one unit N and P. In general, these ratios can be a predictive indicator of nitrogen- and phosphorus-use efficiency in many ecosystems (Zhou et al., 2013). Increased nutrient use efficiency in plants is known to be an important strategy for adaptation to nutrient-poor habitats. Our finding that grazing exclusion increased C:N and C:P ratios and decreased N and P concentrations is not consistent with previous studies on soil nutrient enrichments (Feyisa et al., 2017; Wang T. et al., 2017), thus suggesting that soil nutrient changes induced by grazing exclusion are not the primary reason for changes in plant nutrient strategies. In addition, we found that grazing exclusion significantly increased the C:N and C:P ratios in leaves, stems, and the aboveground parts of *L. chinensis* individuals. This implies that changes in C:N and C:P ratios of plant tissues may be a temporary response to livestock grazing, which differs from the lag effect of plant morphology, community productivity, and soil features.

Nutrient limitation for grassland plants in a plant-herbivore interaction system has long been a controversial topic. The N:P ratio in ecological stoichiometry is often adopted to estimate nutrient limiting factors in plants across many ecosystems (Zhan et al., 2017). A previous study revealed that a N:P ratio > 16 indicates P limitation at a community level, while a N:P ratio < 14 is indicative of N limitation (Bai et al., 2012). We found that N:P in *L. chinensis* individuals was lower than the threshold of 14:1 for N limitation, and that N:P gradually decreased with the increase of grazing exclusion time, especially in *L. chinensis* stems. This may be related to the increasing rapid growth and N requirements of stems with the increase of the duration of grazing exclusion.

Our findings have important implications for understanding the potential effects of grazing on plant traits and nutrient strategies in grassland regions, and for developing useful management practices to improve pasture productivity (Wu et al., 2014). Theoretically, we provide new evidence for understanding the relationship between plant size and nutrients in grazing ecosystems. Practically, grazing-induced changes in individual plant size under increased human activity will dramatically affect pasture productivity, and the effects will be especially profound in the pastures in Inner Mongolia, China (Bai et al., 2012). Based on our findings that there were potentially P and N limitations of the dominated *L. chinensis* in free-grazing and grazing exclusion grasslands respectively, we suggest promoting pasture utilization reasonably and P-optimized management should be preferred in degraded grasslands, whereas N-optimized management should be the primary measure to increase pasture productivity in grazing exclusion pastures.

## Conclusion

We concluded that the increase of grazing exclusion time exponentially increased plant morphological traits, and nutrient concentrations and stoichiometry in the degraded pasture were also strongly impacted. There was a significant negative correlation between the degrees of plasticity and stability of various morphological traits. The patterns of biomass allocation of *L. chinensis* were significantly correlated with the increased duration of grazing exclusion, primarily determined by changes in stem traits. Phenotypic traits were negatively correlated with N and P concentrations, but they were positively correlated with C concentration. Further, we detected that there was a tradeoff between concentrations and the accumulation of N and P, but coordinated variation of C. We discovered that changes in N, P, and C allocation induced by grazing exclusion were derived from biomass partitioning and leaf to stem ratios of nutrient concentrations. In general, our findings suggest that there is a significant negative effect between plant sizes and nutrient traits under grazing exclusion.

## Author Contributions

ZL: wrote the paper and the data analysis. TB: designed the study. JD: carried out the experiments. GY, JS, and XL collected the data and data analysis.

## Conflict of Interest Statement

The authors declare that the research was conducted in the absence of any commercial or financial relationships that could be construed as a potential conflict of interest.
